# *Giardia's* Epithelial Cell Interaction *In Vitro*: Mimicking Asymptomatic Infection?

**DOI:** 10.3389/fcimb.2017.00421

**Published:** 2017-09-26

**Authors:** Martin R. Kraft, Christian Klotz, Roland Bücker, Jörg-Dieter Schulzke, Toni Aebischer

**Affiliations:** ^1^Unit 16 Mycotic and Parasitic Agents and Mycobacteria, Robert Koch-Institute, Berlin, Germany; ^2^Institute of Clinical Physiology, Charité Campus Benjamin Franklin, Berlin, Germany

**Keywords:** Giardia, giardiasis, TEER, barrier function, Caco-2, transwell, permeability

## Abstract

The protozoan parasite *Giardia duodenalis* is responsible for more than 280 million cases of gastrointestinal complaints (“giardiasis”) every year, worldwide. Infections are acquired orally, mostly via uptake of cysts in contaminated drinking water. After transformation into the trophozoite stage, parasites start to colonize the duodenum and upper jejunum where they attach to the intestinal epithelium and replicate vegetatively. Outcome of *Giardia* infections vary between individuals, from self-limiting to chronic, and asymptomatic to severely symptomatic infection, with unspecific gastrointestinal complaints. One proposed mechanism for pathogenesis is the breakdown of intestinal barrier function. This has been studied by analyzing trans-epithelial electric resistances (TEER) or by indicators of epithelial permeability using labeled sugar compounds in *in vitro* cell culture systems, mouse models or human biopsies and epidemiological studies. Here, we discuss the results obtained mainly with epithelial cell models to highlight contradictory findings. We relate published studies to our own findings that suggest a lack of barrier compromising activities of recent *G. duodenalis* isolates of assemblage A, B, and E in a Caco-2 model system. We propose that this epithelial cell model be viewed as mimicking asymptomatic infection. This view will likely lead to a more informative use of the model if emphasis is shifted from aiming to identify *Giardia* virulence factors to defining non-parasite factors that arguably appear to be more decisive for disease.

## Introduction

*Giardia duodenalis* (also *Giardia lamblia* or *Giardia intestinalis*) is an ubiquitous protozoan parasite of the order diplomonadida and forms a species complex of eight different phylogenetic groups (assemblages) characterized by different host specificities. Assemblages A and B are infectious to humans and other mammals (Thompson and Monis, 2012). It is responsible for more than 280 million symptomatic cases of infection, i.e., cases of giardiasis associated with gastrointestinal malfunctions (see below), every year, worldwide. Infections are acquired by uptake of dormant cyst forms of the parasite mostly via contaminated drinking water, and highest prevalences are reported for countries with poor access to higher sanitation standards, though it is one of the most common reported parasitic infections in other countries as well. Since 2004, more attention is paid to this pathogen due to WHO's “Neglected Diseases Initiative” (Adam, 2001; Savioli et al., 2006; Ankarklev et al., 2010). *Giardia* sp. cysts transform into their trophozoite stage after passing the host's stomach and the latter forms colonize the duodenum and upper jejunum where they replicate, attach to the intestinal epithelium using their adhesive disc and feed on luminal nutrients.

## The challenge: symptomatic vs. asymptomatic infections

In the context of this article, we define a symptomatic infection, i.e., giardiasis disease, as being characterized by acute gastrointestinal complaints, like diarrhea, abdominal pain, nausea, and vomiting (Adam, 2001; Ankarklev et al., 2010) and we like to distinguish it from asymptomatic infections defined by the absence of such acute symptoms. Asymptomatic infections according to this definition would include infections that—particularly if recurring—result in malabsorption/malnutrition phenotypes or may represent pure colonization without any pathology, which was recently shown by Garzon et al. (2017) on asymptomatic children, who found no correlation between *Giardia*-infection and barrier dysfunction, but only of barrier dysfunction and wasting and stunting. However, infections may also trigger post-infectious syndromes such as irritable bowel disease (D'Anchino et al., 2002; Wensaas et al., 2012; Hanevik et al., 2014; Litleskare et al., 2015; Halliez et al., 2016; Nakao et al., 2017).

The reasons for these fundamental differences of the outcomes of infections remain unclear (Adam, 2001; Troeger et al., 2007; Klotz and Aebischer, 2015; Tysnes and Robertson, 2015). A mechanism that was proposed to link to acute symptoms was the breakdown of the intestinal barrier function of the epithelium, leading to increased permeability with bacterial invasion as a possible result (Buret, 2007; Ankarklev et al., 2010). This was probably inspired by the impressive *in vitro* phenotypes obtained with certain bacterial, gastrointestinal pathogens and their products/toxins/proteases (Malago et al., 2003; Fajdiga et al., 2006; Rees et al., 2008; Liu et al., 2012; Anderson et al., 2013; Fiorentino et al., 2013) as well as other parasitic protozoans like *Entamoeba histolytica, Cryptosporidium parvum*, or *Blastocystis* sp. (Li et al., 1994; Leroy et al., 2000; Buret et al., 2003; Betanzos et al., 2014; Wu et al., 2014). It is fair to say that this has motivated numerous studies with the implicit goal of identifying a similar functional correlate of acutely symptomatic giardiasis in *in vitro* models. Here, we review *in vitro* investigations that aimed at finding such a robust correlate for acute clinical symptoms and conclude that, frustratingly, this goal was largely missed. We hypothesize that this is because these models reproduce the prevailing inconspicuous course of *G. duodenalis* infection, rather than one that is associated with acute symptoms. We propose, as others did (Bartelt and Sartor, 2015), that non-parasite factors in combination with *G. duodenalis* infection are most likely causing acute symptoms and adapting *in vitro* models to search for such factors should be attempted. We also propose that a number of epithelial functions that may give insight into non-acute but probably more relevant symptoms linked to malabsorption/malnutrition phenotypes have not yet been investigated *in vitro* but should become a focus of future work.

Two studies can serve to illustrate the dilemma that we face when trying to define pathological correlates of *Giardia* infection at the histological/cellular level. One study performed by Oberhuber et al. (1997) analyzed 567 *Giardia*-positive cases identified in a retrospective study of gastrointestinal biopsy samples collected consecutively over an eight-year period in the frame of a gastrointestinal pathology service in Germany. This number of *Giardia* positive cases represented ~0.3% of all samples analyzed in that period. Reasons for soliciting histopathological analyses were described as follows: “most of the patients experienced unspecific gastrointestinal complaints that prompted an upper endoscopy” (Oberhuber et al., 1997). The authors conclude that “the histology of the small-bowel mucosa is inconspicuous in most subjects with giardiasis.” It is reasonable to assume that such a consecutive study on biopsies sampled a random cross-sectional collection of mostly adult patients and therefore, it is justified to conclude that most *Giardia*-infections do not lead to acute symptoms. In contrast, a study focusing on acutely symptomatic cases reported epithelial barrier dysfunction, altered *tight junction* composition, and increased signs of apoptosis (Troeger et al., 2007).

### Epidemiological findings on epithelial dysfunction

There have been several epidemiological studies investigating functional correlates of disease such as gut permeability in different patient collectives suffering from *G. duodenalis* infection. Very recently Rogawski et al. (2017) and Kosek (2017), through a multisite birth-cohort study, showed that persisting infection within the first 6 months of life was correlated with reduced weight and height for age Z scores at 2 years of age. However, this was not dependent on diarrhea, i.e., independent of acutely symptomatic infection. More likely, stunting was the result of changes to gut permeability, assessed by Lactulose (L) and mannitol (M) excretion assays according to which permeability was positively correlated with parasite detection, which agrees with some earlier findings (Dagci et al., 2002; Goto et al., 2002) but contradicts others (Serrander et al., 1984; Campbell et al., 2004; Goto et al., 2009). Even studies using the same method vary in their results. For example, Goto et al. (2002) assessed in a cross-sectional study intestinal parasite infection and permeability of 210 Nepali children aged 0–60 months who were living in poor conditions. Fourteen percent of 173 were acutely infected with *Giardia*. L:M ratios suggested increased permeability in *Giardia*-infected (0.43; *n* = 8) compared to uninfected children (0.25; *n* = 45; *p* = 0,014). However, standard deviations were very high and the stepwise multiple and logistic regression analyses used is known to be prone to type I errors (Burnham and Anderson, 2004). On the other hand, in a later longitudinal study by Goto et al. (2009) the examination of 298 children from Bangladesh until their second year of age and with comparable living conditions did not find a correlation of L:M ratios and *Giardia*-infection. Similar results were found in Gambian children (2–15 months of age) by another longitudinal L:M study by Campbell et al. (2004). In other studies, even a negative correlation of *Giardia*-infection to severe diarrhea could be observed (Bilenko et al., 2004; Kotloff et al., 2013; Muhsen et al., 2014).

Overall, epidemiological evidence for altered permeability as a consequence of frequent *G. duodenalis* infection exists. Effects are weak and appear to be inconsistent probably because data are confounded by non-acute, asymptomatic cases included in studied patient collectives. However, the evidence is rather against *G. duodenalis* infection alone as being a clear cause of acute symptoms such as diarrhea. In view of this data, the quest is open for improved *in vitro* models and respective readout parameters that may link to distinct symptoms, such as changes of permeability, molecular transport, and absorption mechanisms.

### Investigations of epithelial barrier functions *in vitro*

**Figure 1:**
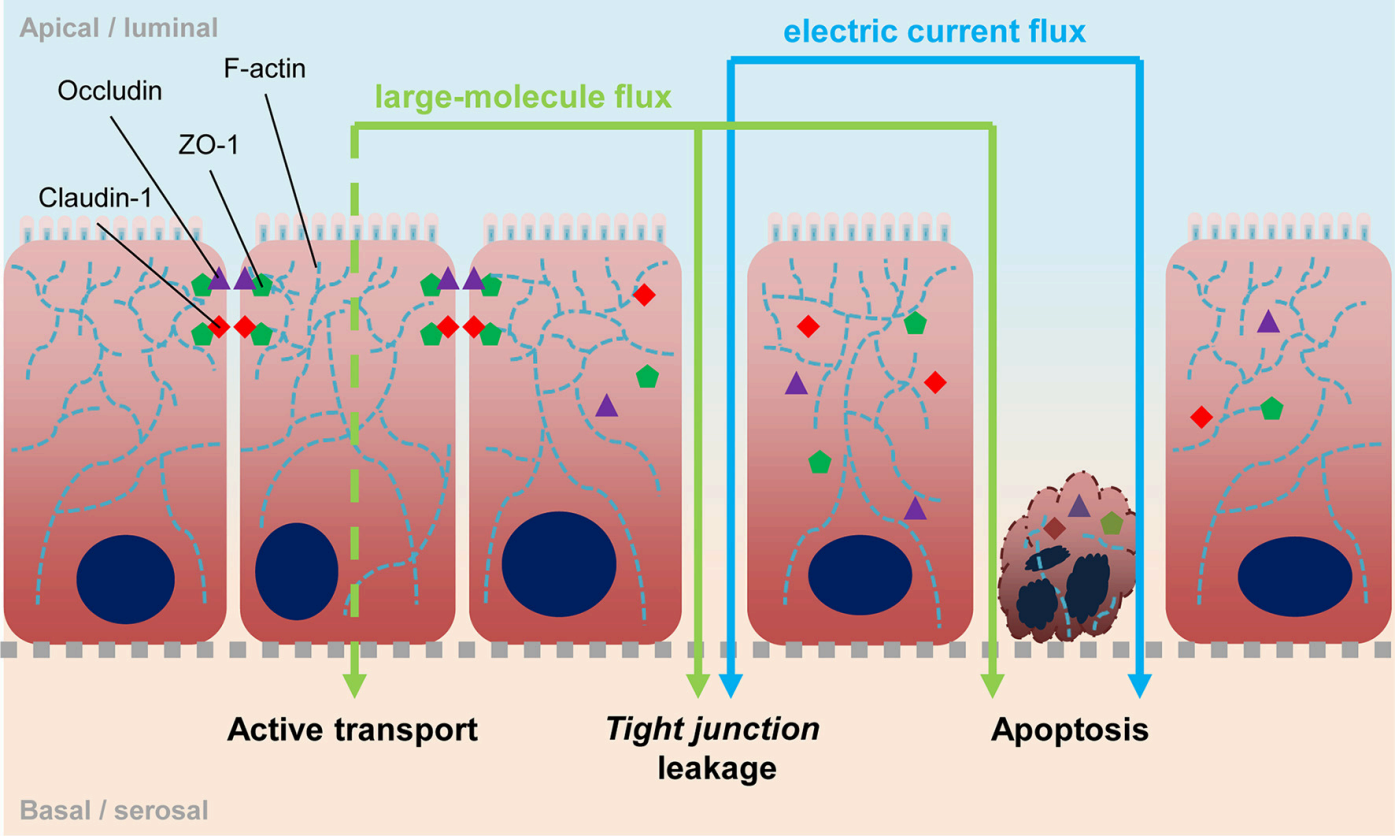
Epithelial barrier. Scheme depicts the epithelia barrier and possible ways of its impairment. *Tight junctions* are located at the lateral sides in apical proximity and consist of membrane-spanning proteins like occludin and different claudins, as well as scaffold proteins like ZO-1 which connect the cytosolic ends of *tight junction* proteins to the actin cytoskeleton. Exchange of those proteins, leading to different *tight junction* compositions, affect specific permeability. Degradation of *tight junctions* is followed by an increase of unspecific permeability. Loss of cellular contacts lead to apoptosis and vice versa, apoptosis induces *tight junction* degradation. With measuring TEER, open breaches in the epithelium can be detected directly. The use of labeled molecules can also detect such leaks, however molecules can also be actively transported transcellularly e.g., via pinocytosis.

Thinking of giardiasis disease as being linked to different syndromes of altered gut barrier function, acute and non-acute, should allow derivation of distinct readout parameters as proxies for the diverse symptoms when interrogating *in vitro* cell culture models with *Giardia* parasites. Current readouts include probing electrophysiological properties of epithelial cell monolayers such as trans-epithelial electric resistance (TEER), assessing transport and permeability changes using tracer molecules, or inferring monolayer integrity from changes of abundance and localization of proteins that make up *tight junctions* (Figure 1). In addition, epithelial tissues also function as communication layers relaying signals to other organs such as the immune system, and cyto- and chemokine responses were also used as readout parameters to characterize the effect of infection.

#### TEER as a surrogate of acute effects on tissue integrity

**Figure 2:**
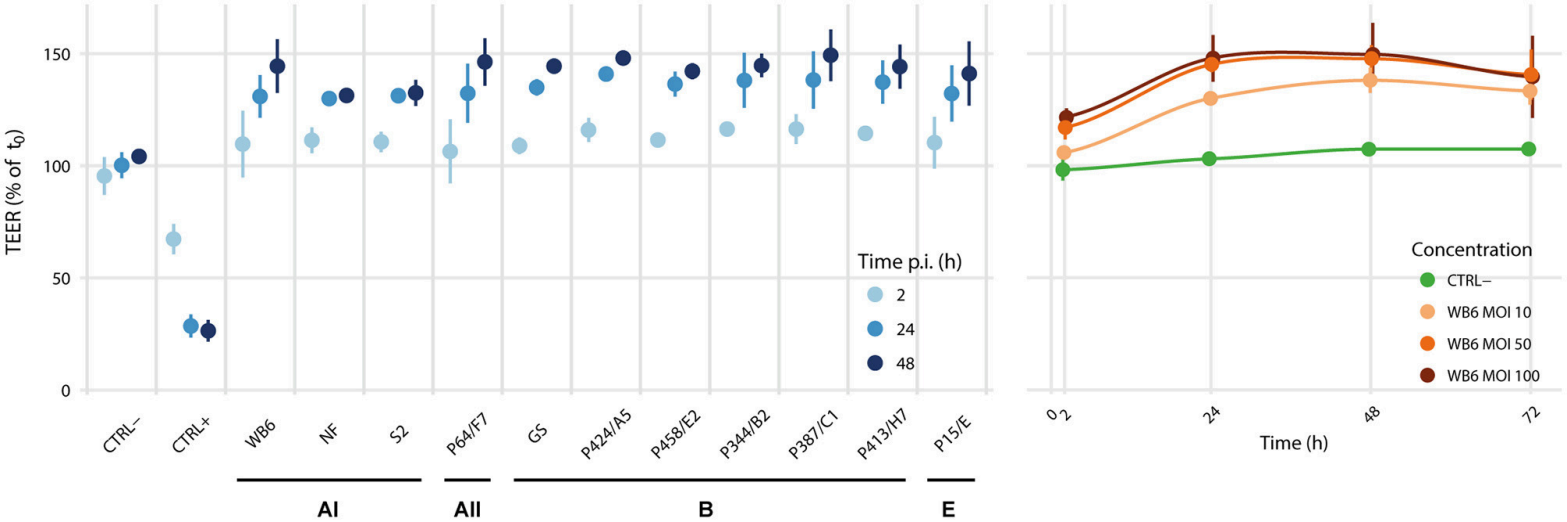
Isolate comparison and dose dependency. We analyzed the effects of *Giardia* colonization of epithelial monolayers on TEER using the Caco-2 clone bbe, since it is reportedly more homogeneous than other clones or its parental line (Sambuy et al., 2005; Liévin-Le Moal, 2013; Srinivasan et al., 2015). Although, Caco-2 cells have been derived from a human colon cancer patient, a 21-day phase of confluent incubation ensures their differentiation toward a polarized small intestinal enterocyte-like cell type, considered as a model for the small intestinal epithelial barrier (ibid.). In this setup, we tested 11 different *G. duodenalis* isolates at MOIs of 20, including 5 reference strains WB6, NF, S2 (assemblage AI), GS (assemblage B), P15 (assemblage E) and 6 newly established clinical isolates (1 assemblage AII and 5 assemblage B). Data is relative to measurements before infection. Uninfected controls (CTRL-) were sham treated, monolayers with induced apoptosis using 1 μM staurosporine served as positive (leaky) controls (CTRL+). Each point represents the mean of 3 independent experiments with 3 monolayers per condition per experiment. Error bars indicate standard deviation. No evidence for parasite-induced decreases in TEER was found. In contrast, all tested isolates led consistently to a dose-dependent TEER-increase, without significant differences between isolates or assemblages.

The measurement of TEER as an indicator for paracellular permeability is a well-accepted method to estimate acute pathophysiological effects on cell barrier function of intestinal epithelial cells (Srinivasan et al., 2015). An important prerequisite for a robust readout is the presence of an electro-physiologically intact monolayer. TEER measurements to investigate the effect of *Giardia* trophozoites on epithelial function have been used by several groups in diverse setups but were implemented with possibly relevant experimental differences (Supplementary Table 1). Our own data (Figure 2) suggest that exposure of Caco-2 cells *in vitro* to diverse *G. duodenalis* isolates derived from samples of symptomatic patients does not affect epithelial monolayer integrity. This is in good agreement with some previous studies (Chavez et al., 1986, 1995; Tysnes and Robertson, 2015) but is in contrast to others (Teoh et al., 2000; Humen et al., 2011; Maia-Brigagao et al., 2012), who described a TEER decrease of up to 45%. If one assumes, as also proposed by others (Bartelt and Sartor, 2015), that *G. duodenalis* infection alone is insufficient to cause acute disease and, therefore, that infections are mostly asymptomatic, then one would not expect an effect on TEER. Since several studies have reported the contrary, however, it seems justified to analyze possible reasons for the discrepancies as they may point toward the currently elusive additional factors that are postulated here and elsewhere (Bartelt and Sartor, 2015) to precipitate acute symptoms.

As listed in Supplementary Table 1, studies differ in experimental detail and it is of interest to analyze whether these could have affected the overall outcome of the respective TEER measurements. For example, one variable of the popular Caco-2 model is that several clonal Caco-2 populations exist and their response can vary according to a particular readout (Katelaris et al., 1995; Sambuy et al., 2005; Liévin-Le Moal, 2013; Srinivasan et al., 2015). Therefore, we tested the parental cell-line and the clonal cell line Caco-2 bbe in a similar setting—with the same result (Supplementary Figures 1A,B). Of note, different subpopulations—also unintentionally established by ongoing passaging of those heterogeneous cells—may explain highly different basic TEER values between setups, which range from ~160 Ωcm^2^ (Teoh et al., 2000) to >1,200 Ωcm^2^ (Scott et al., 2002). In our experiments, Caco-2 (parental) had a basic TEER of ~300 Ωcm^2^, whereas Caco-2 bbe offered initially ~200 Ωcm^2^ (Supplementary Figure 2) but increased this value in a linear manner after 37 passages to 350 Ωcm^2^ at passage #53 (Supplementary Figure 3), which is a known phenomenon (Sambuy et al., 2005; Srinivasan et al., 2015).

Parasite number is another factor that varies between studies, where MOIs of 0.5–8 have been described (Humen et al., 2011). In our experiments, we used MOIs of similar range (1 and 10; Supplementary Figure 1C) as well as very high parasite doses up to MOIs of 100 (Figure 2B or Supplementary Figures 1A,B). Thus, it seems unlikely that parasite:enterocyte ratios can explain conflicting results between studies. Specific characteristics of chosen parasite isolates cannot be excluded, but several identical isolates were tested in more than one study—including our own experiments—with different outcome (Supplementary Table 1). Thus, other differences in experimental setups must be considered.

It has been discussed that trophozoites' physical attachment alone may increase TEER and therefore obscure possible barrier defects (Chavez et al., 1995; Teoh et al., 2000; Chin et al., 2002; Tysnes and Robertson, 2015). However, exposure to the potent *Giardia* detachment reagent formononetin, known to remove trophozoites almost instantly without affecting their viability (Fisher et al., 2013), also did not led to normal TEERs of infected conditions (Supplementary Figure 4A). Though, formononetin-treatment right after infection seem to reduce its magnitude (Supplementary Figure 4B), this contrasts to data by Humen et al. (2011), who noted a TEER decrease which was dependent on trophozoite attachment that could not be triggered by spent medium. However, spent medium and sonicates led to a decline in TEER in other studies (Teoh et al., 2000).

In many studies, infected monolayers were washed intensively with ice cold PBS to remove parasites and subsequently measured TEER (Supplementary Table 1). Our experiences with such washing procedures led to erroneously high TEERs and highly increased variances of measurements, an effect that required several hours of re-incubation to normalize (Supplementary Figure 5). In experiments that do not rely on washing, such as the use of sonicated lysates or trophozoite-conditioned DMEM, Caco-2 monolayers were shown to reduce their TEERs when exposed to, for example, NF or S2 lysates already after 24 h (Teoh et al., 2000). By destroying compartmentalization, lysates may lead to release of factors such as proteases or other enzymes that affect epithelial cell function (Buret, 2007; Cotton et al., 2011, 2014; Bhargava et al., 2015). However, in our hands WB6 lysates and heat-inactivated trophozoites, both corresponding to a MOI of 20, did not influence Caco-2 monolayers' TEER (Supplementary Figure 6A), while filtered apical supernatants from a WB6-MOI-100-72-h condition weakly recapitulated the TEER increase noted in experiments with intact parasites (Supplementary Figure 6B), indicating that products of vital *Giardia* trophozoites, such as products of secreted microvesicles (Evans-Osses et al., 2017) or stressed Caco-2 cells, may be responsible for the observed TEER increase.

Another variation in experimental design is the time of culture and differentiation before infection (Supplementary Table 1). Since monolayers change TEER properties over time of cultivation (Supplementary Figure 2) and less differentiated cells may be more sensitive to disturbing stimuli, findings can be confounded which can explain the disparate results in seemingly similar studies (Teoh et al., 2000; Humen et al., 2011; Maia-Brigagao et al., 2012, and for own data see Supplementary Figure 7).

The medium composition is another important parameter in *Giardia* co-culture experiments. Complete substitution of apical DMEM volume with *Giardia* growth-medium TYI-S-33 can lead to a steep initial TEER increase of monolayers, that can be further accentuated if monolayers are infected with WB6 trophozoites. However, the presence of TYI-S-33 will eventually lead to the collapse of infected and non-infected monolayers (Chavez et al., 1986; Supplementary Figure 8). Thus, takeover of TYI-S-33 components can clearly be a confounder.

Although, presence of O_2_ for a microaerophilic pathogen is of relevance, this parameter does not seem to be decisive either. When aerobic and anaerobic conditions were compared using Caco-2 with WB6 trophozoites, anaerobic conditions showed no TEER decrease within 77 h, but indicated a higher TEER increase in both infected and uninfected Caco-2 monolayers. Prolonged incubation led to barrier failure within 139 h in all anaerobically cultivated Caco-2 monolayers, whereas TEER of aerobic cultivated monolayers remained stable and—for infected conditions—elevated (Supplementary Figure 9). Of note, viability of WB6 in our setups remained high with 57–75% of trophozoites still alive after 48 h and 32–48% after 72 h in unmodified culture conditions (data not shown).

The sodium-dependent glucose cotransporter (SGLT)-1 has been shown to inhibit enterocyte apoptosis in Caco-2 cells under high glucose conditions (Yu et al., 2005, 2008). Therefore, it is possible that depending on the cell culture media applied apoptotic effects of *Giardia* are masked by high glucose concentrations, e.g., in the often-used standard DMEM. However, such an effect is unlikely to have occurred in studies that used the parental Caco-2 since they do not express SGLT-1 in relevant amounts (Turner et al., 1996; Yu et al., 2008). In studies that used the Caco-2 bbe line which express this transporter (Turner et al., 1996) this is difficult to rule out, but in our experiments with the Caco-2 bbe line and low glucose conditions, comparable to Yu et al. did not affect TEER values when compared with cultures using normal DMEM (Supplementary Figure 10).

Serum is another possible component, capable of masking apoptosis induction, since it is known to contain corresponding inhibitors (Zoellner et al., 1996). In our experiments without FBS, however, no significant differences regarding *Giardia*-infection were found (Supplementary Figure 7). In contrast, the magnitude of effects of staurosporine which was used as a apoptosis-inducing control on TEER was clearly affected by FBS as predicted (Zoellner et al., 1996).

Another interesting confounder may be the *Giardia lamblia* virus (GLV). It is described as a double-stranded RNA virus of 7 kb size and belongs to the family *Totiviridae* (Wang and Wang, 1986; Janssen et al., 2015). GLV can infect several but not all *Giardia* isolates (Miller et al., 1988), depending on the expression of a specific surface receptor (Sepp et al., 1994). The related *Leishmania*-specific endosymbiont “*Leishmania* RNA virus-1” (LRV1) is known to affect the severity of leishmaniasis (Ives et al., 2011). Likewise, GLV could also influence *Giardia*'s virulence, though no correlation regarding GLV infestation of *Giardia* isolates to symptomatic or asymptomatic patients were found in the past (Jonckheere and Gordts, 1987). Our experiments—at least regarding TEER—also suggest no detectable differences between GLV-infected or uninfected WB6 and GS trophozoites on Caco-2 monolayers (Supplementary Figure 11).

In summary, variations in experimental design due to the lack of standardization confound the search for robust effects on TEER induced by *G. duodenalis*. The current data in particular of the Caco-2 *in vitro* co-culture model rather suggests that *Giardia* alone does not induce acute barrier-defects.

#### Alteration in *Tight junction* composition

Intestinal epithelial barrier function is highly dependent on cytoskeleton architecture and *tight junction* composition (Hidalgo et al., 1989; Troeger et al., 2007). *Giardia*-induced changes in the F-actin cytoskeleton were observed after incubation with sonicates (Scott et al., 2002), with living trophozoites, sonicates and spent medium (Teoh et al., 2000) or only with attached trophozoites (Humen et al., 2011). All these studies found also alterations to respective TEER values (Supplementary Table 1). *Tight junction* proteins, ZO-1 and claudin-1 showed in some studies altered localization, that could be prevented by various treatments, e.g., by EGF- (Buret et al., 2002) or caspase-3 inhibitor treatment (Chin et al., 2002), or MLCK-inhibitor addition (Scott et al., 2002; see summary in Supplementary Table 1). *Ex vivo*-analysis of epithelial tissue from symptomatic patients, showed 30% reduced claudin-1 abundance (Troeger et al., 2007), which was not evident in any *in vitro* study. On a technical note, all *in vitro* studies which showed alterations of the *tight junction* complex, experiments were conducted on plastic (chamber slides) or glass (coverslips) support which alter Caco-2 monolayer morphologies (Supplementary Figure 12).

**Figure 3:**
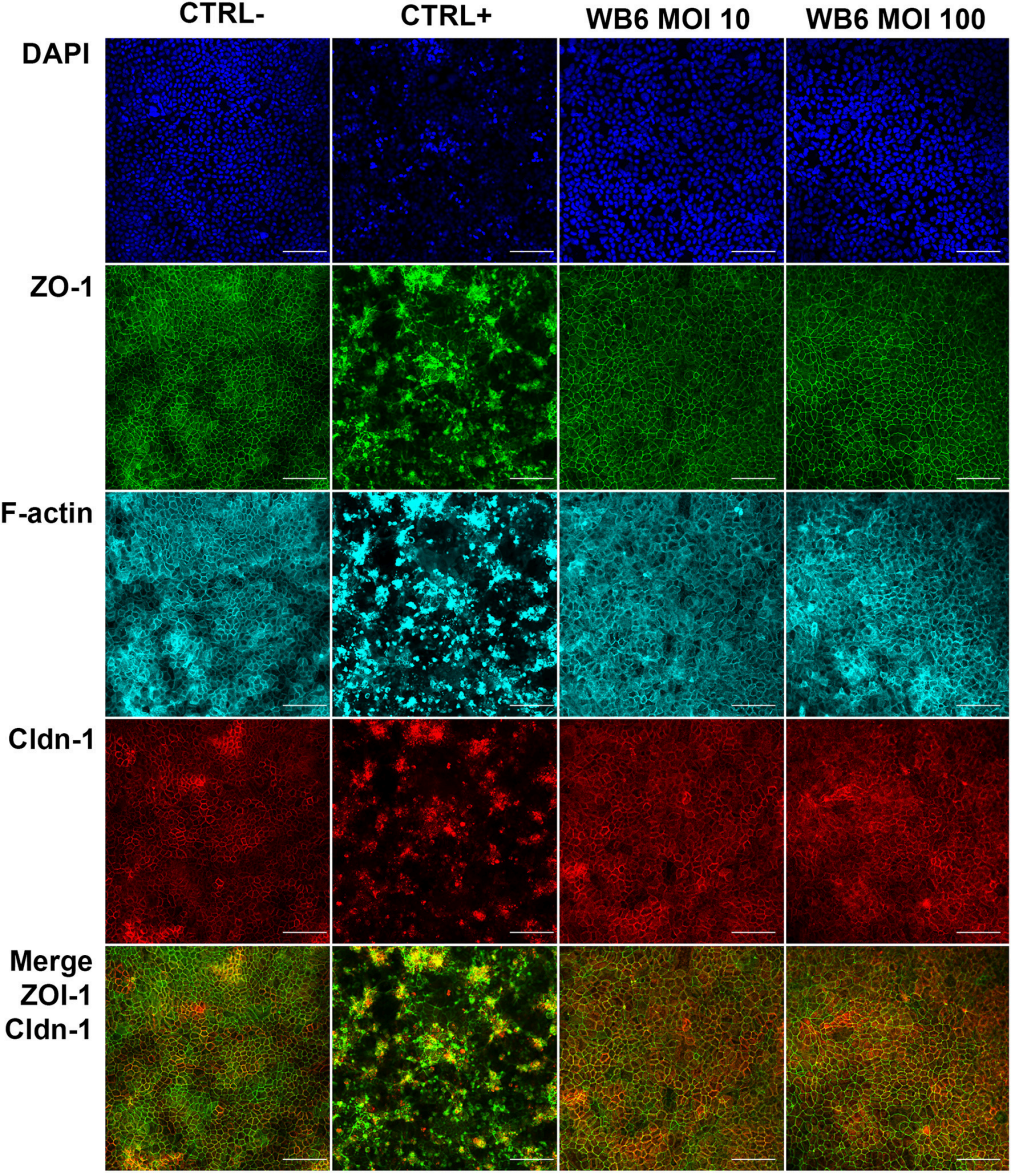
*Tight junction* integrity. Micrographs depict *tight junction* proteins ZO-1 (green), claudin-1 (red), and F-actin cytoskeleton (cyan) after 72 h, comparing uninfected control (CTRL−), 1 μM staurosporine control (CTRL+), and *G. duodenalis* isolate WB6 of MOI 10 and 100. Scale bars indicate 100 μm. Nuclear staining with DAPI is shown in blue. No significant changes in any of the observed proteins due to *Giardia*-infection are detectable.

**Figure 4:**
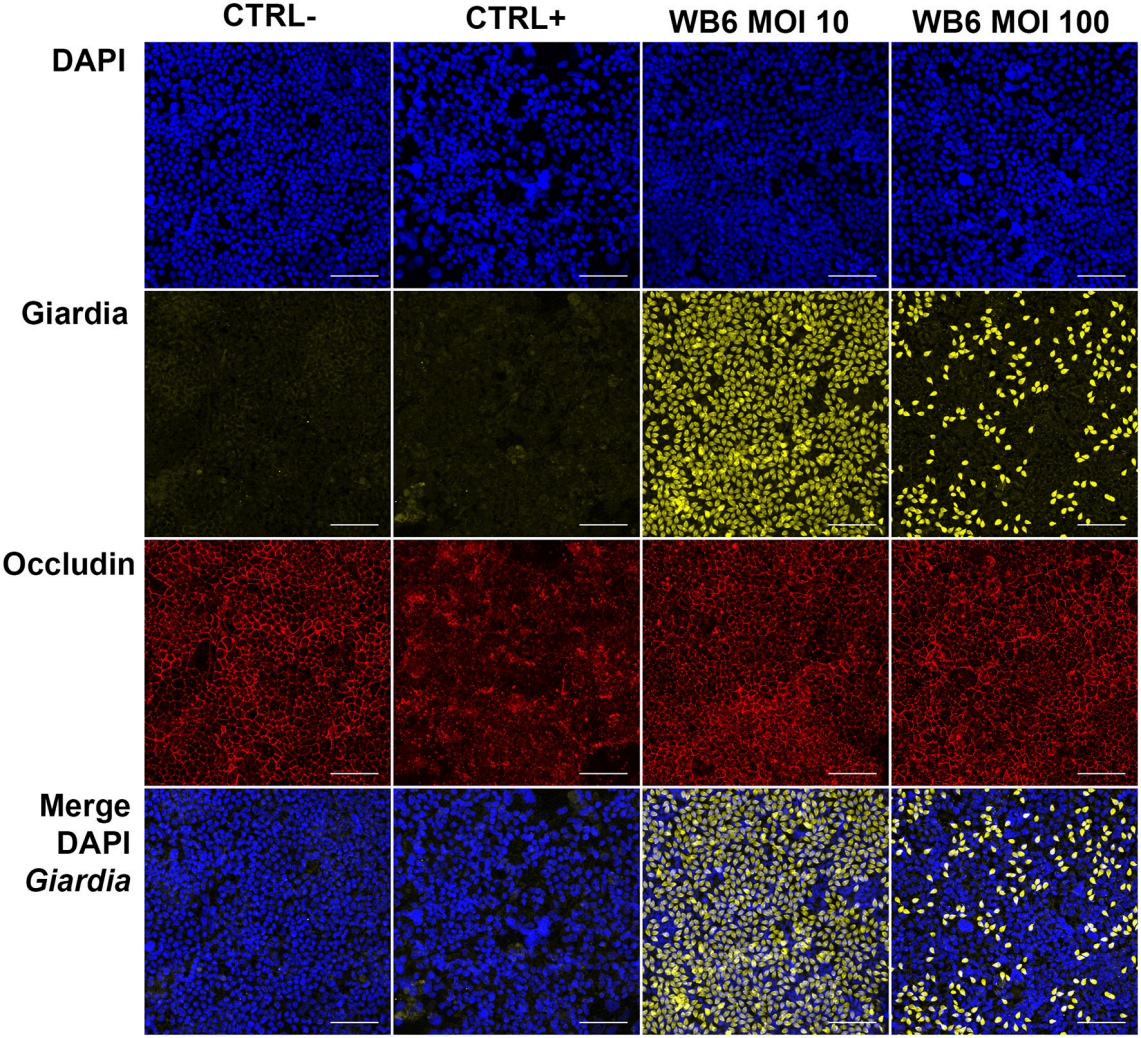
*Tight junction* integrity and trophozoites. Micrographs depict *tight junction* protein occludin (red) and trophozoites (yellow) in Caco-2 bbe monolayer after 24 h, comparing uninfected control (CTRL-), 1 μM staurosporine control (CTRL+) and *G. duodenalis* isolate WB6 of MOI 10 and 100. Scale bars indicate 100 μm. Nuclear staining with DAPI is shown in blue. No significant changes concerning occludin due to *Giardia*-infection are detectable. A MOI of 10 is sufficient to completely cover the Caco-2 monolayer. Of note, MOI 100 show artificially less trophozoites attached than MOI 10 condition, possibly due to rapid nutrient consumption followed by starvation and subsequent detachment of the trophozoites.

**Figure 5:**
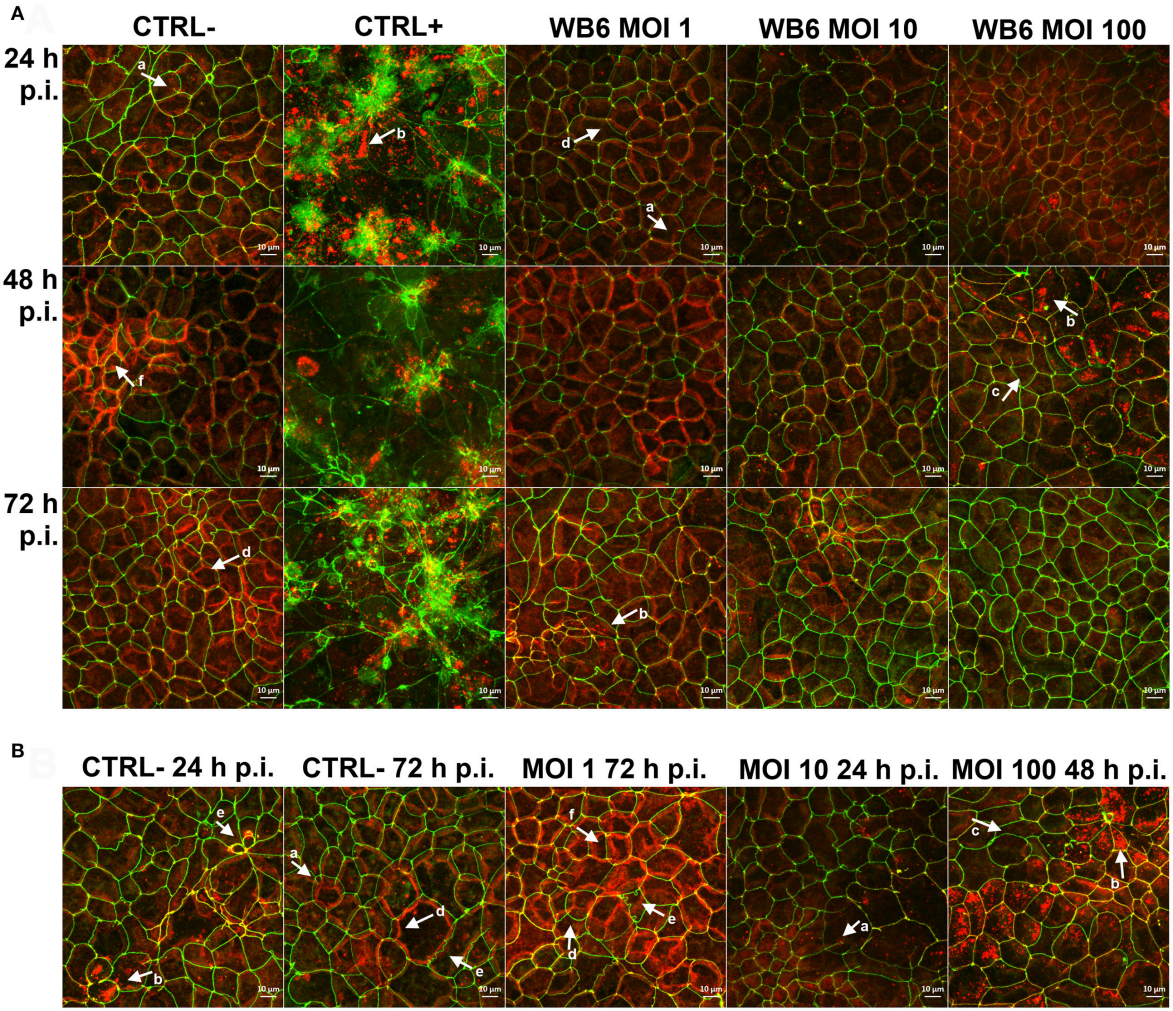
Effects on *tight junction* proteins. Micrographs are derived from z-stacks, spanning whole cells, by using maximum intensity projection and depict *tight junction* proteins ZO-1 (green) and claudin-1 (red) in Caco-2 bbe monolayer, comparing uninfected control (CTRL−), 1 μM staurosporine control (CTRL+) and *G. duodenalis* isolate WB6 of MOI 1, 10 and 100 after 24, 48, and 72 h **(A)**. An additional selection of micrographs of same samples is shown in **(B)**, illustrating Caco-2 peculiarities. Arrows in **(A,B)** indicate observed random phenotypes like ZO-1 “branching” (a), claudin-1 flocculation and delocalization (b) or its absence directly next to it (c), mismatch of ZO-1 and claudin-1 localization (d), odd cellular contacts (e), and areas of increased claudin-1 presence (f).

In contrast, but in agreement with the TEER measurements, no significant differences between infected or uninfected monolayers with respect to F-actin and *tight junction* proteins were observed on electrophysiologically tight filter-supported Caco-2 layers (Figures 3, 4). Occasionally, effects as described in other studies, were detectable but lacked any correlation with infection (Figure 5). Due to heterogeneity of Caco-2 monolayers leading to a mosaic of microvilli formation (Katelaris et al., 1995; Sambuy et al., 2005; Liévin-Le Moal, 2013), the reported *G. duodenalis* induced microvilli depletion (Chavez et al., 1986, 1995; Buret et al., 1990, 1991) that should manifest as apical cytoskeletal changes could have been missed since changes reported were not drastic.

In conclusion, integrity of the *tight junction* protein complex also suggests an asymptomatic interaction of *Giardia* alone with the Caco-2 model. Alterations described by others may be related to growth in non-transwell systems. Of note, described disruptive effects on ZO-1 have been described as secondary to apoptosis and not directly induced by the parasite (Chin et al., 2002; Buret et al., 2003; Bojarski et al., 2004; Zehendner et al., 2011).

#### Epithelial barrier dysfunction due to apoptosis

Apoptosis is a tightly regulated process in the gut epithelium. For giardiasis, reported rates vary greatly between studies from no changes to controls (Chavez et al., 1986, 1995; Katelaris et al., 1995; Maia-Brigagao et al., 2012; Tysnes and Robertson, 2015, and own findings), to minor increases from 1 to 1.5% in symptomatic patients' mucosa (Troeger et al., 2007), to isolate specific effects of sonicates (Chin et al., 2002), to significant increases up to 41% with a rather low MOI of 3 after just 16 h using HCT-8 cells (Panaro et al., 2007) or only significant after long term co-culture (Fisher et al., 2013). HCT-8 cells were also shown to undergo increased apoptosis in mixed isolate infections (Koh et al., 2013); a finding that we could not reproduce with Caco-2 cells using NF and S2 (data not shown). Thus, effects on apoptosis also seem to be highly dependent on experimental setup and lack the robustness to allow a clear conclusion.

### Tissue permeability as a surrogate of altered molecular fluxes

Absorption and trans-epithelial transport of biomolecules are key functions of the gut epithelia (Kiela and Ghishan, 2016) and permeation assays can be used to probe these functions.

#### Labeled compounds as marker for paracellular permeability

A number of *in vitro* studies assessed paracellular permeability using fluorescein isothiocyanate (FITC)-conjugated dextran. Buret et al. (2002) noted a more than 40-fold increase of the trans-epithelial FITC-dextran flux in co-cultures with *Giardia* trophozoites. This effect was associated with alterations on *tight junction* protein distributions (Buret et al., 2002) and could be abolished when monolayers were pretreated with epidermal growth factor (EGF). Since bovine EGF can act on human cells, and since serum used in co-culture experiments provides several growth factors with overlapping effects, differences between serum batches must be considered as potential confounders. Similar results in a comparable setting were also reported using sonicates of various *Giardia* isolates (WB, PB, NF, and S2; all assemblage A; Chin et al., 2002) and on SCBN monolayers (Scott et al., 2002). Interestingly, large molecule permeability could be abolished using Myosin light-chain kinase (MLCK)-Inhibitor ML-9 (Scott et al., 2002). The mechanism proposed implicates phosphorylation of Myosin light-chain (MLC) by MLCK activity that leads to alterations in the F-actin and ZO-1 composition and eventually to increased permeability. An *ex vivo* study using FITC-dextran in *Giardia* GS infected mice compared effects on FITC-dextran permeability on day 7 (colonization phase) and 35 (post-clearance phase) after infection and found a slight increase in permeability at both time points of ~30% that was correlated with cleavage of occludin and also increased endocytosis of bacteria (Chen et al., 2013). Our findings using FITC-dextran correlate with our respective TEER measurements (Supplementary Figure 13), showing no increased permeability of *Giardia*-infected monolayers, but of apoptosis-induced controls. Thus, like before the *in vitro* findings are as contradictory—also with respect to this parameter—as the *in vivo* studies mentioned before.

#### Combined assessment of para- and transcellular flux

It is known, at least for *in vivo* studies, that individual sugars alone are not a reliable indicator of intestinal permeability and therefore disaccharide/monosaccharide ratios should be used instead (Johnston et al., 2000). Lactulose and mannitol for example are not transported via active monosaccharide transport systems but due to their difference in size can serve as a marker for transcellular permeability through channels (mannitol) and paracellular permeability, or *tight junction* leakage (lactulose; Andre et al., 1988). However, L:M ratios are not used very often *in vitro*, but other compounds had been used instead to assess and distinguish paracellular and transcellular flux. Using horse radish peroxidase (HRP) and creatinine as trans- and paracellular markers, respectively, infection with both a lab adapted and a field isolate, led to increased mucosal to serosal transcellular flux. Also paracellular permeability was increased by the field isolate (R-2), which is contradictory to the observed TEER increase in the same study (Tysnes and Robertson, 2015). Another study by Hardin et al. (1997) on *Giardia* S2*-*infected Mongolian gerbils could not find increased permeability to [^51^Cr]EDTA (permeability similar to lactulose), but macromolecular transport of BSA was induced. A study on rats also suggest *Giardia*'s interference with active transport, but the other way around: Glucose and glycine absorption was decreased, whereas potassium which diffuses passively through the epithelium was unaffected (Anand et al., 1980). However, one should keep in mind that malabsorption could be either a result of impaired uptake due to dissipated osmotic and ion gradients originated from leaky *tight junctions* and apoptotic cells, or due to a reduction in the absorptive area because of villus shortening and microvilli depletion. The latter, a reduction in absorptive area, has been shown to be one of the more consistently found pathological features of *Giardia* (Chavez et al., 1986, 1995; Buret et al., 1990, 1991; Troeger et al., 2007) and could explain some study discrepancies.

Results with Caco-2 cells in permeability assays have to be interpreted with caution as these cells are known to actively and passively transport contents and are extensively used in pharmaceutical drug absorption assays (Hidalgo et al., 1989; Artursson et al., 2001; Sambuy et al., 2005; Sun et al., 2008). When cultivated on plastic surfaces and not on transwell-filters, these cells tend to form large liquid-filled vacuole-like structures (Supplementary Figure 12) probably because they are not able to route those volumes through to a basally located compartment. Also, osmotically active marker substances, like sugars, can confound results by interfering with osmotic gradients. Enhanced active transport by *Giardia*, however, is understudied, but, should become more of a focus also because antigens or whole microbes of the intestinal lumen can be transported and be of pathological relevance. Indeed, Chen et al. (2013) found evidence for enhanced endocytosis of bacteria, rendering such a scenario possible.

### Analysis of chemokine/cytokine profiles in *Giardia* epithelial cell co-cultures

**Figure 6:**
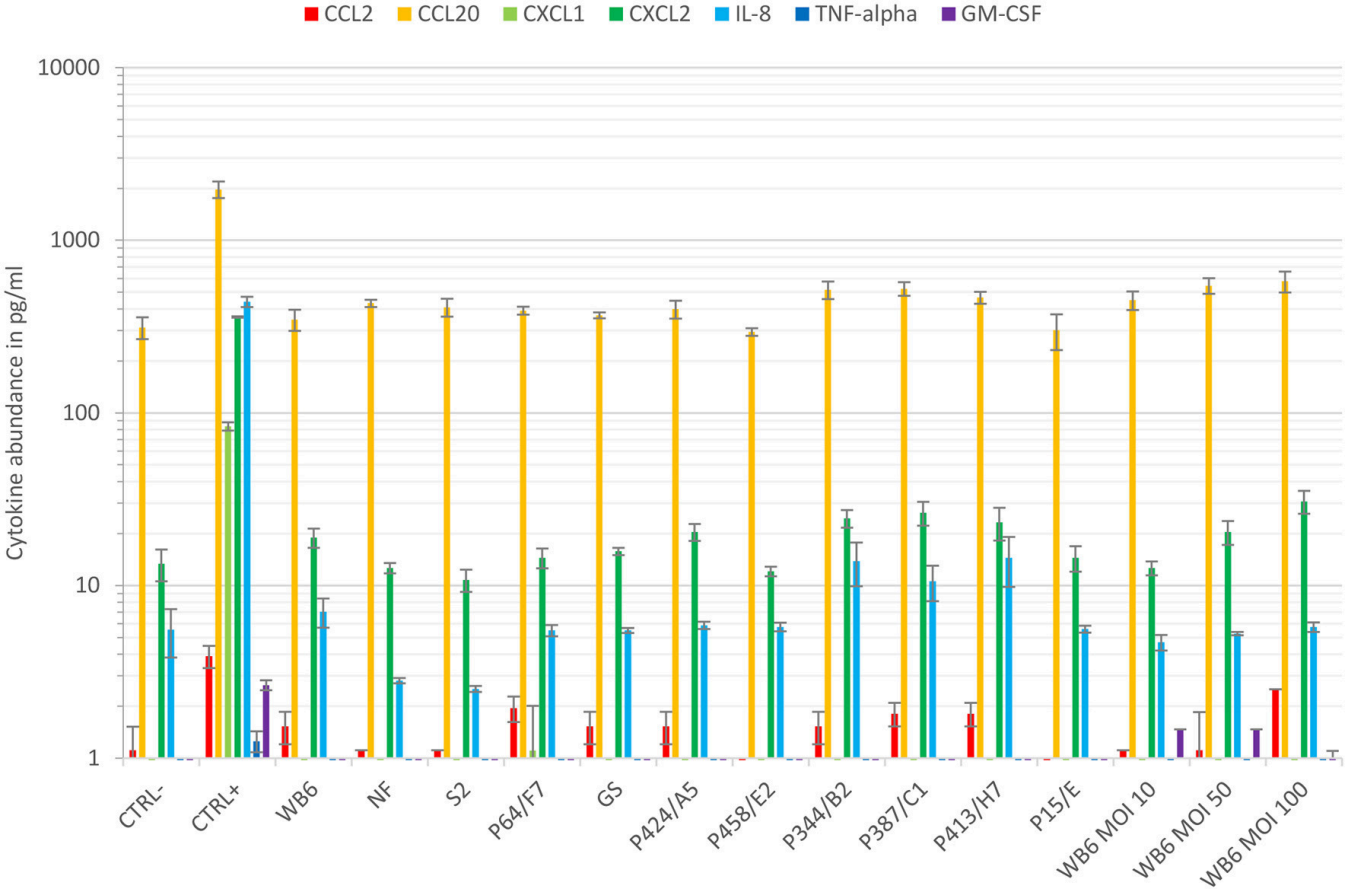
Cytokine response. Cytokine levels of CCL2, CCL20, CXCL1, CXCL2, IL-8, TNFα, and GM-CSF were compared using basal supernatants of the conducted 48–72 h TEER experiments and the Luminex® technology. Data show means and standard deviation of cytokine levels of supernatants, gathered from at least two independent TEER experiments. Except for CCL20, cytokines were barely detected in this compartment independent of infection. In contrast, monolayers insulted with staurosporine (CTRL+) increased amounts of CCL20, CXCL1/2, and IL-8, whereas CCL2, TNFα, and GM-CSF remained below the lower quantification limit.

In order to validate the asymptomatic outcome of our Caco-2 setup further, several chemokines/cytokines potentially or claimed to be upregulated during giardiasis were investigated. Most of the cytokine analyses that have been published were performed using cell monolayers grown on cell culture plastic. A seminal study used micro-array analysis of *Giardia*-infected Caco-2 cells during the first 18 h and showed an increase in CCL20, CCL2, and CXCL1/2 mRNA abundance among others (Roxström-Lindquist et al., 2005). However, increased IL-8 and TNFα mRNAs were not detected. Another study indicated no changes to IL-8, CCL2, GM-CSF, and TNFα (Jung et al., 1995). Hence, it appears that responses to *Giardia* infections differ from the generalized response by enterocytes of colonic origin to bacterial infections (Jung et al., 1995). This was corroborated by Fisher et al. (2013) for CCL2, GRO-isoforms (CXCL1/2), or IL-8 in the Caco-2 co-culture system. Interestingly, Caco-2 co-cultured with macrophages alone elicited GRO-isoform and IL-8 expression but this was abolished again when *Giardia* trophozoites were added, indicating immune-modulatory capabilities of the parasite (Fisher et al., 2013). *Giardia*'s ability to alter immune responses has been described by others, too (Kamda and Singer, 2009; Cotton et al., 2014, 2015). Our data using the Caco-2 transwell culture system also suggest no triggering of basolateral release of CCL2/20, CXCL1/2, IL-8, TNFα, or GM-CSF by different isolates or MOIs tested (Figure 6). Overall, our cytokine data also suggest an asymptomatic *Giardia*-infection. Whether this may be due to parasites' immune-modulatory features (Kamda and Singer, 2009; Cotton et al., 2014, 2015) requires further studies.

## Conclusion

As mentioned in the introduction, one of the major enigmas in giardiasis is what distinguishes acutely symptomatic from asymptomatic outcomes of *Giardia*-infections. In this work, we reviewed and discussed current *in vitro* epithelial cell culture systems that we propose do reproduce asymptomatic host-parasite interaction rather than acutely symptomatic giardiasis. We propose for further development of these systems a structured approach that aims at identifying appropriate *in vitro* correlates for distinct clinical symptoms: The search for correlates of acute manifestations such as diarrhea could focus on readouts such as TEER, *tight junction* function, and ion secretion, but should focus on testing combinations of parasite and non-parasite factors. Food factors, or the lack of certain nutrients, could determine trophozoite virulence as well as bile or pancreatic secretions. Especially bile is a requirement for axenic trophozoite cultivation (Keister, 1983; Halliday et al., 1988) and it could stimulate the parasite not only to grow, but also to increase its virulence. It is also imaginable that certain host proteases could cleave *Giardia* proteins in a way that an inactive virulence function is activated, similar to what has been shown as a necessity for host cell entry of influenza virus particles (Kido et al., 2012). Of note, *Giardia* specifically decreases the activity of pancreatic trypsin, but not of chymotrypsin (Seow et al., 1993), which suggests that certain proteases can be hazardous for *Giardia* and may disturb a finely balanced silent infection to provoke pathogenic reactions. Additionally, a host's genetic background linked to hypogammaglobulinemia, IgA deficiency or cystic fibrosis or a host's phenotype comprising reduced gastric acidity, stress, co-infections, or other concomitant diseases, could contribute to the pathogenesis (DuPont, 2013). Furthermore, since the mechanisms of trans-epithelial transport are as diverse as the respective substrates (Kiela and Ghishan, 2016) and our lack of knowledge if and how *Giardia* affects those transport systems, the search for correlates and explanations for more protracted effects such as stunted growth that may be linked to altered fluxes of particular biomolecules, should focus on a systematic assessment of epithelial cell functions related to those transcellular transport and absorption mechanisms. Another important factor relevant for the symptomatic outcome may be the host's microbiome, as suggested by others (Singer and Nash, 2000; Chen et al., 2013; Slapeta et al., 2015). The fact that young children after the lactation period are generally more affected by *Giardia* infestation than older children or adults may be an epidemiological correlate of a not-yet-settled microbiome that is more susceptible to interference by the parasite. Moreover, differences in food resources and cultural habits, resulting in distinct intestinal bacterial colonization may underlay the variances noted between studies that cover different geographic regions. Additionally, very recent publications reporting experimental findings also point toward an increasing role of the intestinal microbiota during *Giardia* infections (Allain et al., 2017; Barash et al, 2017; Beatty et al., 2017). Finally, since giardiasis resembles symptoms of food allergies, hypersensitivity or intolerance like coeliac disease, pathogenesis could be more related to the host's individual immune reaction (Scott et al., 2004). This might explain why *Giardia*-infections are less severe or even protective in developing countries, where allergic diseases are usually not as prevalent. However, due to the parasite's ability to enhance sensitization toward food antigens (Di Prisco et al., 1998), the causal relationship will be difficult to investigate. Moreover, the general *in vitro* methodology of *Giardia*-host interaction studies requires better standardization with the goal to offer robust, inter-laboratory evaluated models.

## Author contributions

All authors contributed equally to design and conception of this work. MK conducted research on literature and collected experimental data. CK, RB, JS, and TA contributed to experimental design and intellectual input. RB and JS helped interpreting data and MK, CK, and TA contributed to the manuscript.

### Conflict of interest statement

The authors declare that the research was conducted in the absence of any commercial or financial relationships that could be construed as a potential conflict of interest.
