# Supplementary material for: Giardia's Epithelial Cell Interaction In Vitro: Mimicking Asymptomatic Infection?
Source: Front Cell Infect Microbiol. 2017 Sep 26;7:421. doi: 10.3389/fcimb.2017.00421 (PMC5622925; doi:10.3389/fcimb.2017.00421)
Supplement: Supplementary file 1 [file DataSheet1.docx]

Supplementary Material

*Giardia’s* epithelial cell interaction in vitro: mimicking asymptomatic infection?

Martin R. Kraft^1,2*^, Christian Klotz^1^, Roland Bücker^2^, Jörg-Dieter Schulzke^2^, Toni Aebischer^1*^

^1^Unit 16 Mycotic and Parasitic Agents and Mycobacteria, Robert Koch-Institute, Berlin, Germany

^2^Institute of Clinical Physiology, Charité Campus Benjamin Franklin, Berlin, Germany

*** Correspondence:**Martin R. Kraft
[KraftM@rki.de](mailto:KraftM@rki.de)

Toni Aebischer
[AebischerA@rki.de](mailto:AebischerA@rki.de)

# Material and Methods

## Cell culture

Caco-2 bbe cells, obtained from the lab of Prof. Dr. Jörg-Dieter Schulzke, and the Caco-2 parental cell line (herein also referred to as “Caco-2 (standard)”), obtained from the American Type Culture Collection (ATCC®, Manassas, VA, USA) were used with focus on bbe clone, since it is more homogeneous and described as the best immortal cell line to model the intestinal epithelium. Cell lines were *Mycoplasma* sp. negative and grown in T25 cell culture flasks (TPP®, Schaffhausen, Switzerland) with high glucose Dulbecco's modified Eagle's medium (DMEM GlutaMAX™; Gibco™; Paisley, Scotland, GB), supplemented with 10% foetal bovine serum (FBS; Sigma-Aldrich / Gibco™), 10 mM HEPES, 100 µg/mL streptomycin, 100 U/mL penicillin and 1x non-essential amino acids (MEM NEAA; Gibco™; Paisley, Scotland, GB). Cultures were incubated at 37°C in a humidified atmosphere of 5% CO_2_ and passaged weekly with TrypLE™ Express (Gibco™; Grand Island, NY, USA). Experiments span passage numbers between 5 to 11 (standard) and 14 to 54 (bbe).

## *Giardia* parasites

*G. duodenalis* parasites of assemblage A1 (WB6; NF; S2), A2 (P64/F7), B (GS; P424/A5; P458/E2; P344/B2; P387/C1; P413/H7, and the livestock-specific assemblage E (P15/E) of the institution’s biobank were cultivated in Keister’s modified TYI-S-33 medium, supplemented with 10% fetal or adult bovine serum (FBS; Sigma-Aldrich / Gibco™; ABS; Gibco™), 100 µg/mL streptomycin, 100 U/mL penicillin and 0,05% bovine bile. WB6 and GS are well known isolates, used by laboratories since decades. They are representatives for assemblage A and B isolates, respectively. NF (from a water-isolate during a giardiasis outbreak in Newfoundland) and S2 (isolated from sheep) are both a gift from the lab of Andre Buret. Those had been described in prior papers to be more virulent than WB6 (Hardin et al. 1997; Teoh et al. 2000; Chin et al., 2002; Scott et al. 2002). P15/E has been isolated from a pig and is known to be virulent in hoofed animals, predominantly livestock, but cannot infect humans (Jerlström-Hultqvist et al., 2010). All other isolates were derived recently from symptomatic patients. Therefore, the isolates used represent a broad sampling of culturable Giardia isolates derived from clinically manifest infections. Cultures were grown in 10 ml flat-sided tubes (Nunclon™ Δ, Rochester, NY, USA) at 37°C and passaged 3 times a week by placing the tubes into ice water for 20 min to release attached trophozoites. Subsequently tubes were inverted several times and aliquots corresponding to the isolates growth rate were sub cultured. One day prior experimental starts, cultures were prepared to harvest trophozoites of the log-phase.

## Transwell setup, TEER and FITC-dextran permeability

100000 cells of Caco-2 (standard) or Caco-2 bbe were seeded into 0.6 mm² transwell PCF filter inserts of 0.4 µm pore size (Millicell®, Merck Millipore; Darmstadt, Germany), fitted into wells of a 12-well plate (TPP®, Schaffhausen, Switzerland) with 400 µl DMEM in the apical and 1200 µl in the basal compartment. Development of confluency was monitored with TEER measurements before every medium exchange, two times a week. In order to avoid disturbance of the forming monolayer, filters were treated in a way that weight forces of the medium push only apically. Cells differentiated for 21-22 days. IFAs suggest final cell numbers per filter ranging from 120000 to 240000. For MOI calculations, 200000 cells per filter were assumed. 2-4 h prior experiments medium was exchanged and trophozoites were collected by chilling on ice and centrifugation at 1000 x*g* for 5 min, washed two times with PBS(-/-), counted in a disposable Neubauer counting chamber (C-Chip, Biochrom AG; Berlin, Germany) and resuspended in pre-heated DMEM corresponding to respective MOIs. Lysates were created by French press of $1\times{10}^{7}$ trophozoites/ml DMEM (continuous cycles at 4°C). Trophozoite solutions or lysates were added into the apical compartment and volumes were equalized to 500 µl with DMEM in all filter inserts. Positive controls received a final concentration of 0.5-1 µM of the apoptosis-inducing agent staurosporine in the basal compartment with subsequent, gently mixing via pipetting. Conditions ran in triplicates, except for some controls (2 filters usually for untreated controls, 1 filter for staurosporine control). TEER measurements were conducted on a 38°C heating block (since TEER is dependent on temperature) with an ERS-2 Voltohmmeter (Millicell®, Merck Millipore; Darmstadt, Germany) with an electrode in the apical and an electrode in the basal compartment avoiding direct physical contact with the monolayer, mean basic resistance of 12 empty filters in DMEM were subtracted from raw data (Ω) for blank correction and surface were corrected (0.6 cm²) to calculate Ωcm². Relative changes to CTRL- as well as longitudinal changes were also assessed.
For molecule-permeability tests, FITC-dextran (MW 3000) has been pipetted into the apical transwell volume in a final concentration of 1 mg/ml at 24 h p.i. and incubated for 2 h at 37°C. Afterwards 50 µl of the basal compartment has been taken and filled up to 200 µl with PBS in a 96-well plate. Fluorescence intensity has been measured using a TECAN Infinite 200 PRO plate reader at 485 nm excitation and 525 nm emission (20 nm bandwidth). Raw data was blank- and volume-corrected and expressed as percentage of the fluorescence intensity of samples from the apical control compartment.

## Immunofluorescence assays

After final TEER measurement, monolayers were immediately fixed with 4% PFA at RT for 15 min or precipitated in -20°C methanol for 20 min (only for occludin-stainings). Monolayers were washed three times with PBS containing MgCl_2_ and CaCl_2_ (PBS(+/+); Gibco™; Paisley, Scotland, GB) with subsequent permeabilization and blocking in a solution containing 0,2% Triton-X and 1% BSA in PBS(+/+) for 1.5 h at RT. Monolayers were washed again with PBS(+/+) and 100 µl of primary antibody solutions, diluted in PBS(+/+) with 1%BSA, were apically applied (monoclonal mouse anti human ZO-1 1:1000, 610967 BD Biosciences; polyclonal rabbit anti human claudin-1 1:300, 71-7800 Invitrogen; polyclonal rabbit anti human occludin 1:1000, 40-6100 Invitrogen) and incubated for 1 h at 37°C. After three washing steps with PBS(+/+), secondary antibodies (DTAF-conjugated goat anti-mouse 1:500, 115-016-003 Jackson ImmunoResearch Laboratories Inc.; Cy5-conjugated goat anit-rabbit 1:500, 111-175-144 Jackson ImmunoResearch Laboratories Inc.; Cy3-conjugated Troph-o-Glo undiluted; A900 Waterborne™ Inc.) or phalloidin (Alexa Fluor® 546-conjugated phalloidin 300U 1:300, A22283 Invitrogen) were applied in the same manner. Nuclear staining with Hoechst 33342 (1:1000 of 2 mg/ml stock; H1399 Invitrogen) were conducted for 15 min at RT in the dark with two subsequent PBS(+/+) washing steps. Afterwards, filters were shortly washed in pure H_2_O for desaltation as well as very shortly in 80% ethanol for fast-drying. Filters were cut with a disposable scalpel (Scalpel #11, Fine Science Tools, Heidelberg, Germany) from their frames and embedded in mounting medium (Fluoromount-G®; Southern Biotech, Birmingham, AL, USA) on glass slides under rectangular coverslips (Carl Roth GmbH + Co. KG, Karlsruhe, Germany) for confocal laser scanning microscopy (LSM780; Zeiss, Oberkochen, Germany).

## Cytokine response (Luminex® Assay)

At the end of TEER experiments, samples of the medium in the basal compartment were collected and replicates of conditions within one experiment were pooled and frozen at -80°C for later investigation of cytokine responses. Using the bead-based ELISA system, Luminex® Assay (LXSAH; R&D Systems, Minneapolis, MN, USA), cytokine levels of CCL2, CCL20, CXCL1, CXCL2, IL-8, TNFα and GM-CSF which accumulated until the end of TEER experiments for 48 h (isolate comparison) or 72 h (MOI comparison), were assessed. Samples were diluted 1:2 as stated in the manual and 3 (isolate comparison) or 2 (MOI comparison) samples from independent experiments were used. The assay was conducted according manufacturer’s instructions on Bio-Plex® 200 plate reader system (Bio-Rad; Hercules, CA, USA).

## IT

For plain calculations and diagrams Microsoft® Excel 2010, for statistical analysis R, running one-way ANOVA and Tukey's test with package ggplot2 for graphics was used. Zeiss’ Zen 2012 software (Zeiss, Oberkochen, Germany) to create IF micrographs and Bio-Plex Manager™ software (Bio-Rad; Hercules, CA, USA) to assess Luminex® data were used.

# Supplementary Data

**Supplementary Figure S.1 Concentration and cell line-dependency.** TEER changes of Caco-2 bbe (A, C) and Caco-2 parental line (B) monolayer, relative to measurements before infection, are shown when infected with MOIs ranging from 1-100, as well as uninfected (CTRL-) and treated with apoptosis-inducing agent staurosporine at 0.5 (A, B) -1 (C) µM (CTRL+) for up to 48-52 h. No *Giardia*-linked TEER decreases were detectable.

**Supplementary Figure S.2 Monolayer formation.** Development of absolute TEER changes of 12 individual Caco-2 bbe monolayers, are shown with measurements, taken right before medium exchanges as a matter of routine at day 3, 7, 10, 15, 17 and 21 after seeding of 100.000 cells into transwell filters. After a steep increase in TEER when reaching confluence, what is referred in the literature as “peak resistance”, TEER of monolayers normalize in the subsequent days until reaching a plateau phase. To that time point, Caco-2 cells are regarded as fully differentiated. Conducted experiments started at day 22 after seeding (except for S.7).

**Supplementary Figure S.3 Basic TEER rise with passages.** TEER values of Caco-2 bbe after 21-days of differentiation but before infection or experimental start are shown. After 37 (weekly) passages, cells tend to increase their basic TEER with every further passage in an apparent linear manner. Each data point indicates mean & standard deviation (blue, bold) and median & range (red, thin) of 12 monolayers. Linear regression is shown in green.

**Supplementary Figure S.4 Attachment-dependency.** TEER changes of Caco-2 bbe monolayer, relative to measurements before infection, are shown when infected with assemblage A trophozoites (MOI 20) and treated with 20 µM *Giardia* sp. detachment reagent formononetin (NF+S2 is a mixture of both isolates and is sham treated) after 48 h for 10 min at 37°C and additional 10 min at RT (A), as well as treatment right after beginning or after 24 h until 68 h (B). CTRL+ is 1 µM staurosporine. Formononetin and therefore *Giardia* de- or attachment has no significant influence on the observed TEER increases.

**Supplementary Figure S.5 Effects of cold PBS washing steps.** TEER changes of Caco-2 bbe monolayer, relative to measurements before infection, are shown for intact single filters in the setup of experiment S.6B. Filters were washed 5 times with ice cold PBS(+/+) directly after the 72 h measurement (time scale on x-axis not true beyond this point). TEERs have been measured at the last washing step in PBS(+/+) (90 h value), in DMEM directly afterwards (100 h value), 30 min after incubation at 37°C (105 h value) and 1 (110), 2 (120) and 6 (160) h after incubation. A1, B1 and C1 belong to the former untreated controls and they show the highest variance peaking from 400 to 1400 Ωcm². Furthermore, it shows that monolayers require several hours to normalize after the washing procedure. Therefore, we do not recommend cold washing steps for TEER studies.

**Supplementary Figure S.6. Trophozoite/factor-dependency.** TEER changes of Caco-2 bbe monolayer, relative to measurements before infection, are shown when infected with vital WB6, lysates or dead but intact (heat-inactivated) trophozoites, corresponding to MOI 20 (A), as well as spent medium (filtered supernatants of the apical compartments from uninfected or WB6 MOI 100 conditions of former experiments) or a lysate (B). CTRL+ is 1 µM staurosporine. Only vital trophozoites significantly increase TEER, apical supernatants of 72 h infected monolayers may also rise to a certain degree, though experimental setup is difficult and susceptible to artifacts, as indicated by the increased values of the control.

**Supplementary Figure S.7 FBS-dependency and 7-day after seeding.** TEER changes of Caco-2 bbe monolayer are shown in absolute numbers after just 7 days of incubation instead of the usual 21 days of differentiation. Most monolayers reached peak resistance (see also Figure S.2) but some of them were not yet fully confluent (e.g. CTRL- and one of the WB6 triplicates without FBS). Infection of not fully differentiated monolayers does not seem to lead to different outcomes. Also, the presence of FBS does not influence TEER, except for the staurosporine control, which offered an even more drastic decline without FBS.

**Supplementary Figure S.8. Medium dependency.** TEER changes of Caco-2 bbe monolayer, relative to measurements before infection, are shown when infected with WB6 (MOI 20) under complete (A) and 50% (B) apical substitution of DMEM with TYI-S-33 *Giardia* growth medium. CTRL+ is 1 µM staurosporine. TEER increases with 100% TYI-S-33 alone and even more when also infected with WB6, but monolayer collapse at day 2, irrespective of their infection status. Of note, 100% TYI-S-33 conditions even decrease TEER below staurosporine control, probably due to the higher ion concentration of this medium, resulting in increased conductivity. A 50% substitution leads to an elevated but stable TEER up to 70 h, which rises linear in the presence of WB6.

**Supplementary Figure S.9 O_2_-dependency.** TEER changes of Caco-2 bbe monolayer, relative to measurements before infection, are shown when infected with WB6 (MOI 20) under aerobic and anaerobic conditions. CTRL+ is 1 µM staurosporine. No TEER decreases were detectable under aerobic conditions up to almost 6 days. Generally, TEER increased under anaerobic conditions but collapsed beyond 72 h, irrespective of infection status.

**Supplementary Figure S.10 Glucose-dependency.** TEER changes of Caco-2 bbe monolayer, relative to measurements before infection, are shown when infected with WB6 (MOI 20) under high glucose (normal; 25 mM) and low glucose (5 mM + 20 mM D-Mannitol for osmotic balance) conditions. CTRL+ is 1 µM staurosporine. No significant differences in TEER regarding glucose concentrations were detectable.

**Supplementary Figure S.11 Impact of Giardia virus.** TEER changes of Caco-2 bbe monolayer, relative to measurements before infection, are shown when infected with GLV-positive and negative WB6 and GS trophozoites (MOI 20) conditions. Poly(I:C) (10 mg/ml) serves as control for dsRNA-viruses. CTRL+ is 1 µM staurosporine. No significant differences in TEER regarding GLV presence were detectable. Of note, none of the isolates in Fig. 2A were infected with GLV.

**Supplementary Figure S.12 is not included in this file, because it contains a video format. It can be found in file “Presentation 1”.**

**Supplementary Figure S.13 FITC-dextran permeability.** Flow-through of FITC-dextran to the basal compartment has been measured after 2 h of apical incubation at 24 h p.i. and expressed as apical percentage. Selected conditions of several experiments are shown. GLV = Giardia lamblia virus, p.c. = post confluence (7-day monolayers). Data corresponds to TEER values, showing only increased permeability of monolayers incubated with 1 µM staurosporine.
