# Supplementary material for: Giardia's Epithelial Cell Interaction In Vitro: Mimicking Asymptomatic Infection?
Source: Front Cell Infect Microbiol. 2017 Sep 26;7:421. doi: 10.3389/fcimb.2017.00421 (PMC5622925; doi:10.3389/fcimb.2017.00421)
Supplement: Supplementary file 3 [file Presentation1.PPTX]

## Slide 1
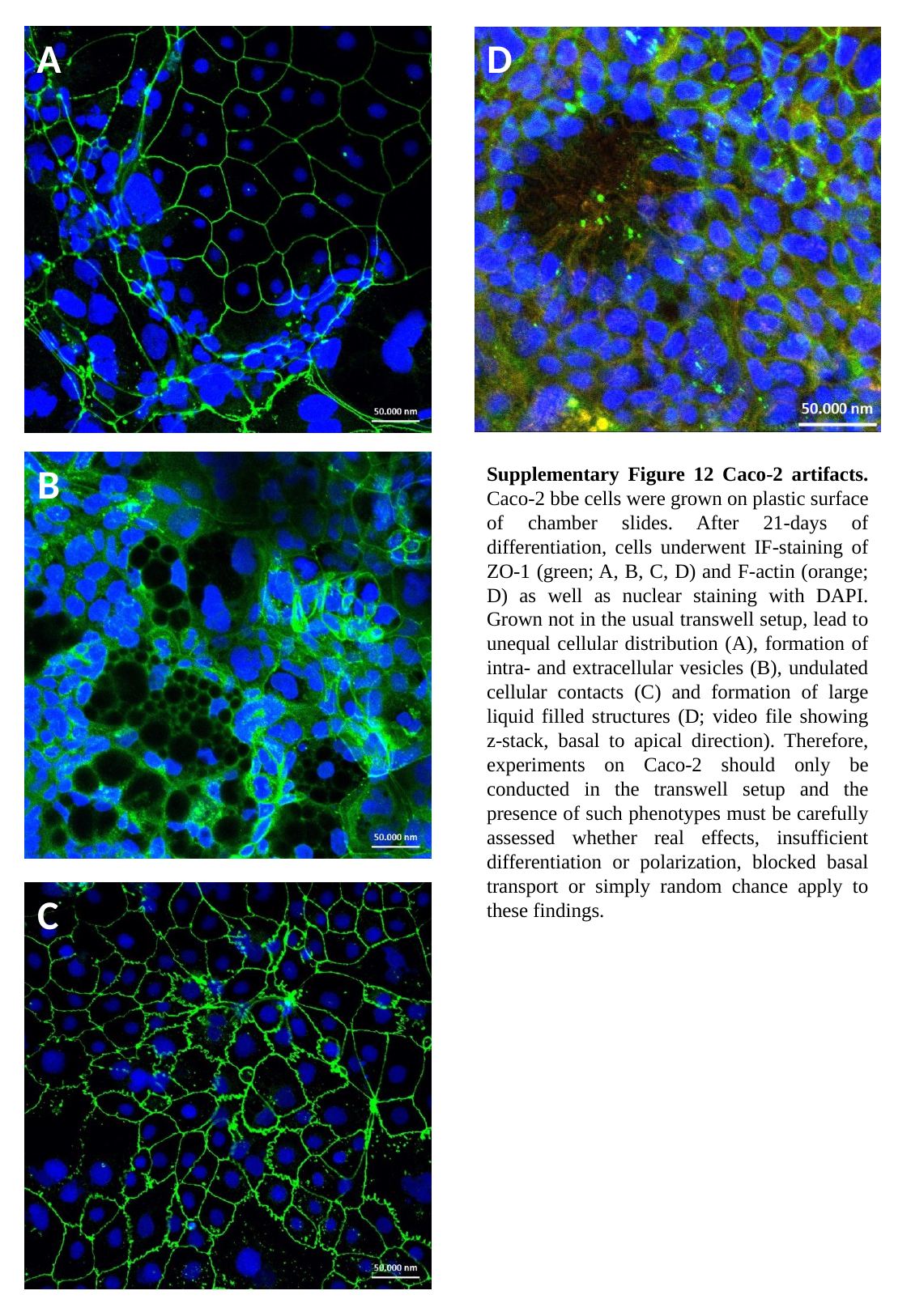

D
A
B
Supplementary Figure 12 Caco-2 artifacts. Caco-2 bbe cells were grown on plastic surface of chamber slides. After 21-days of differentiation, cells underwent IF-staining of ZO-1 (green; A, B, C, D) and F-actin (orange; D) as well as nuclear staining with DAPI. Grown not in the usual transwell setup, lead to unequal cellular distribution (A), formation of intra- and extracellular vesicles (B), undulated cellular contacts (C) and formation of large liquid filled structures (D; video file showing z-stack, basal to apical direction). Therefore, experiments on Caco-2 should only be conducted in the transwell setup and the presence of such phenotypes must be carefully assessed whether real effects, insufficient differentiation or polarization, blocked basal transport or simply random chance apply to these findings.
C
